# Thymoquinone ameliorates acrylamide-induced reproductive toxicity in female rats: An experimental study

**DOI:** 10.18502/ijrm.v21i1.12668

**Published:** 2023-02-08

**Authors:** Maryam AL-ghamdi, Etimad Huwait, Nagwa Elsawi, Soad Shaker Ali, Ahmed Sayed

**Affiliations:** ^1^Department of Biochemistry, Faculty of Science, King AbdulAziz University, Jeddah, Saudi Arabia.; ^2^Experimental Biochemistry Unit, King Fahad Medical Research Centre, King AbdulAziz University, Jeddah, Saudi Arabia.; ^3^Vitamin D Pharmacogenomics Research Group, King AbdulAziz University, Saudi Arabia.; ^4^Department of Chemistry, Lab Biochemistry, Faculty of Science, Sohag University, Sohag, Egypt.; ^5^Faculty of Medicine, Merit University, Sohag, Egypt.; ^6^Biochemistry Laboratory, Faculty of Science, Chemistry Department, Assiut University, Assiut, Egypt.

**Keywords:** Acrylamide, Thymoquinone, Rats, Oxidative stress, Cyclooxygenase 2, Inflammation.

## Abstract

**Background:**

Acrylamide (AA) is a carcinogenic compound that causes severe reproductive impairments and represents a high environmental risk factor. Thymoquinone (TQ) has a unique antioxidant activity and has been widely used as a protective agent against various types of toxicity.

**Objective:**

To evaluate the protective effects of TQ against AA-induced reproductive toxicity in female rats.

**Materials and Methods:**

In this experimental study, 40 female albino rats (120-150 gr, 8-10 wk) were sorted into 4 groups, (n = 10/each), vehicle group (received a daily oral administration of 0.5 ml saline [9%]); AA group (received a daily oral administration with freshly prepared AA, 20 mg/kg body weight) for 21 days which is less than the lethal dose LD ${}_{50}$ of AA in rats (20 mg/kg body weight); AA+TQ group (received a daily oral administration of TQ, 10 mg/kg body weight) after AA intoxication for 21 days, and TQ group (received a daily oral administration of TQ only, 10 mg/kg body weight) for 21 consecutive days. Reproductive hormones, carcinogenic biomarkers, and oxidative stress markers were measured. The histological assessment showed the protective effect of TQ against AA-induced ovarian injury. Network pharmacology analysis and molecular docking approach were carried out to determine the binding affinity of TQ with cyclooxygenase 2.

**Results:**

TQ administration significantly enhanced the functional capacity of the ovary at hormones, oxidative biomarkers, and tumor markers at a significant level of p < 0.001. Besides, TQ protects the ovary of AA-treated rats from the severe degeneration effect.

**Conclusion:**

TQ showed a promising protective effect against AA-induced reproductive toxicity in female rats.

## 1. Introduction

Human reproductive function impairment has increased dramatically in the last few decades (1). This biological issue has raised concerns about the food additives and chemicals that are most common in our environment. Acrylamide (AA) is a vinyl monomer that exists in the form of a white crystalline powder (2). AA is formed naturally during the cooking of starchy foods at high temperatures (120 C). Also, it is found in small amounts in heat-treated food items, cigarette smoke, and adhesives, and is widely used in the biological laboratory (3). Therefore, we are vulnerable to AA risks in our daily life; for example, AA showed a severe neurotoxic effect on animals and humans (4). Besides, it exerts mutagenic and carcinogenic effects on experimental animals (5).Accumulating evidence revealed that AA exposure increases oxidative stress levels in both cells and tissues. Furthermore, cytochrome P450 2E1 is responsible for the biotransformation of AA into glycidamide, which has a higher reactivity toward DNA and proteins, including hemoglobin, than AA itself (6). AA has shown a severe degeneration of ovaries and testicular epithelial tissue in rats (7). Also, AA can cross the placenta and exhibit adverse effects on the development and postnatal effects in the offspring of rodents (8).

Alternative medicine (herbs) has recently gained popularity as potential therapeutic agents and/or for alleviating toxicant and chemotherapy side effects (9, 10). Among these herbs, *Nigella sativa* is an herbaceous plant that has been used as a natural antioxidant. Thymoquinone (TQ) is the major bioactive component of *Nigella sativa.* TQ has a wide spectrum of biological and therapeutic activities as an antioxidant, anti-inflammatory (11, 12), antiapoptotic (13), antitussive (14), antihypertensive (15), antidiabetic (16), antibacterial (17), and anticancer (18). Because of its free radical scavenging activity, TQ has significant antioxidant potential (14). TQ's unique quinine structural feature enables easy access to subcellular compartments, thus enhancing the antioxidant activity and facilitating the ROS scavenging effect (19). TQ exhibited a spermioprotective effect against testes damage (20-22). TQ significantly reduced cysts formation and enhanced ovarian follicles to undergo normal folliculogenesis (23). Also, the dual supplementation of vitamin E and black seed oil enhances folliculogenesis in the ovary of female mice (24).

The female reproductive system is regulated by estrogen, progesterone, follicle-stimulating hormone, and luteinizing hormone (25). However, no sufficient studies evaluate the protective effects of TQ on AA-induced impairments. Therefore, the present work was conducted to assess the antioxidant activity of TQ against AA-induced hormonal disorders in adult female albino rats. Besides, we utilized different bioinformatics approaches to investigate the potential molecular mechanism of the protective effect of TQ.

## 2. Material and Methods

### Chemicals

AA, ≥ 98.0% (GC), CAS: 79-06-1, MW: 71.08 g/mol, mp: 81-87 C, and P Code: 101601204 were obtained from SIGMA-ALDRICH chemical Co (USA). TQ, ≥ 98.0%, CAS: 490-91-5, C ${}_{10}$ H ${}_{17}$ O ${}_{7}$ , MW: 164.20 g/mol, mp: 45-47 C (lit.), b.p: 230-232 C (lit.) and P Code: 1002373178 were obtained from SIGMA-ALDRICH Chemical Co (USA).

### Animals and study design

The experiment was performed on 40 healthy female Wistar albino rats (120-150 gr, 8-10 wk). Animals were provided by the animal house in (Assiut, Egypt). Rats were kept in the experimental room for 2 wk before starting the experiment for acclimatization. They were kept in metal cages under hygienic conditions with free access to a standard rat diet. The animals were sorted into 4 groups, (N = 10), vehicle group (received a daily oral administration of 0.5 ml saline (9%); AA group (received a daily oral administration with freshly prepared AA, 20 mg/kg body weight) for 21 days accordingly (26); AA+TQ group (received a daily oral administration of TQ, 10 mg/kg body weight) after AA intoxication for 21 days accordingly (27), and TQ group (received a daily oral administration of TQ only, 10 mg/kg body weight) for 21 consecutive days.

### Biochemical study

Serum was collected from blood samples taken from the heart of deeply ether anesthetized animals in plain tubes and centrifuged at 5000 rpmfor 10 min, stored at -20 C till analysis. The biochemical tests included female reproductive hormones, and antioxidant activity was performed using commercial kits following the manufacturer's instructions. The kite for reproductive hormone assessment (estradiol, progesterone, follicle-stimulating hormone [FSH], luteinizing hormone [LH], cancer antigen 125 [CA125], and carcinoembryonic antigen [CEA]) was obtained from (CALBIOTECH kit by Calbiotch A Life Science Company, the Egyptian company). The antioxidant activity kit was obtained from (a diagnostic kit by Biodiagnostic Company, the Egyptian company).

### Histological study: Preparation of ovary section and histopathological examination

The ovaries were collected and fixed in 10% neutral-buffered formalin, then the paraffin blocks were prepared. Blocks were sectioned at 4-5 µm thickness by microtome and stained with hematoxylin and eosin stain (Thermo Fisher Scientific, USA) following the standard method. The sections were then examined blindly, and ovary damage was observed under a light microscope at 100 × and 400 × magnifications (Leica, Germany).

### TQ target proteins prediction and molecular docking with cyclooxygenase 2 (COX-2)

A stitch server (http://stitch.embl.de/) was used to predict the interacting target proteins with TQ. A high confidence degree ≥ 0.700 was utilized for the prediction as a cut-off value for the interaction results. Autodock vina 1.5.6 was used to carry out the molecular docking study (28). The 3-dimensional structure of COX-2 with its inhibitor (rofecoxib) was downloaded from a protein data bank (PDB ID: 5KIR) (29). All the water molecules and ligands were deleted from the PDB file and PDBQT file was generated for the docking study. TQ's binding affinity with COX-2 was calculated using the average of the lowest energy of docking. Chimera 1.12 software was used to analyze and visualize the best-scored conformation of the docked model.

### Ethical considerations

Procedures related to the animals were approved by Sohag Institutional Animal Care and Use Committee, Sohag, Egypt (Code: Sohag. 02.01.2021.01).

### Statistical analysis

Graph pad prism software (San Diego, CA. USA) was used to perform all the Statistical analyses. One-way analysis of variables (ANOVA) was used, followed by a post hoc test. All data were expressed as mean ± standard error of the mean (SEM), and the significance level between groups were *p < 0.05, **p < 0.01, ***p < 0.001.

## 3. Results

### TQ ameliorates reproductive hormone levels in AA-intoxicated female rats

**Figure 1:**
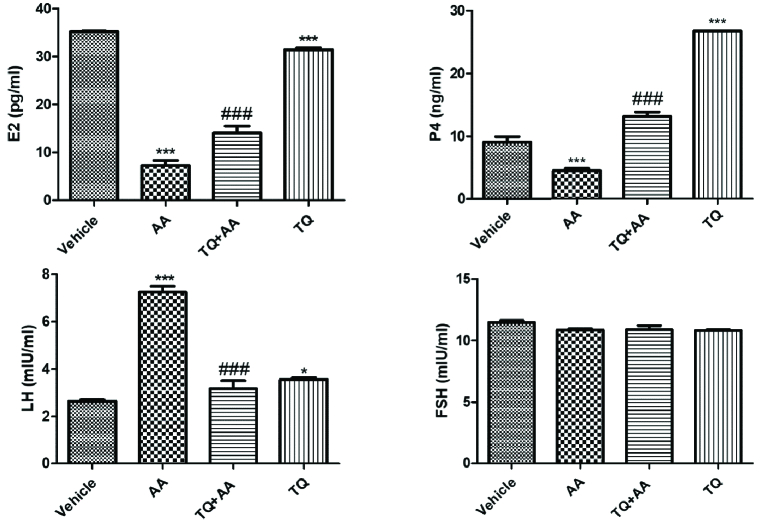
Effect of thymoquinone (TQ) on serum levels of estradiol (E2), progesterone (P4), luteinizing hormone (LH), and follicle-stimulating hormone (FSH). Data represent the Mean ± SEM from 10 female rats. ${}^{*}$ P < 0.05 and ${}^{***}$ P < 0.001 vs. vehicle and ${}^{\#\#\#}$ P < 0.001 vs. acrylamide (AA).

The ameliorative effect of TQ on AA-intoxicated female rats was assessed by determining estradiol, LH, FSH, and serum progesterone, as shown in figure 1. Estradiol and progesterone levels were reduced significantly in AA-treated rats while LH concentration increased, and there was no significant change in FSH levels. On the other hand, TQ administration significantly elevated estradiol and progesterone levels, and there were no significant differences in LH and FSH with respect to the vehicle group.

### CA125 and CEA analysis 

**Figure 2:**
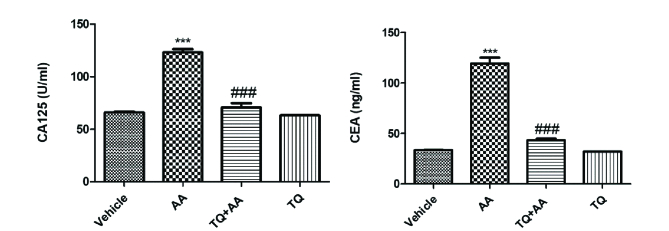
Effect of thymoquinone (TQ) on cancer biomarkers (cancer antigen 125 (CA125), and carcinoembryonic antigen (CEA)) of AA-treated rats. Data represent the Mean ± SEM from 10 female rats. ${}^{***}$ P < 0.001 vs. vehicle and ${}^{\#\#\#}$ P < 0.001 vs. acrylamide (AA).

To evaluate the protective effect of TQ against the carcinogenic effect of AA on ovaries, CA125 and tumor marker CEA were detected, as shown in figure 2. The CA125 (U/L) and CEA (ng/ml) analysis revealed that the mean values of the 2 parameters in G2 were significantly higher (p < 0.001) than in the vehicle group. However, in G3 (AA + TQ), these 2 parameters were significantly lower (p < 0.001) than in G2, and the values were restored to the vehicle group, indicating that TQ protects against AA-induced reproductive impairment.

###  Effect of TQ on oxidant/antioxidant status

**Figure 3:**
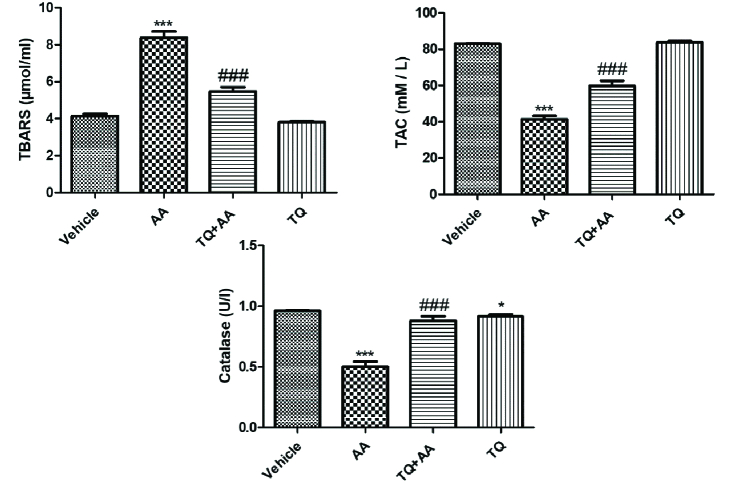
Effect of thymoquinone (TQ) on oxidative injury biomarkers (thiobarbituric acid reactive substances (TBARS), catalase, and total antioxidant capacity (TAC)) of AA-induced reproductive system impairment. Data represent the Mean ± SEM from 10 female rats. ${}^{*}$ P < 0.05 and ${}^{***}$ P < 0.001 vs. vehicle and ${}^{\#\#\#}$ P < 0.001 vs. acrylamide (AA).

To evaluate the protective effect of TQ on the oxidative stress markers, we measured thiobarbituric acid reactive substances (TBARS), catalase, and total antioxidant capacity (TAC), as illustrated in figure 3. TBARS levels elevated significantly, while the catalase and TAC contents were significantly reduced in the rats that received AA alone. AA-TQ-treated rats showed a significant reduction in TBARS content compared to the AA group. In contrast, catalase and TAC contents were increased significantly compared to the AA group. These results highlighted the protective activity of TQ against AA-induced oxidative stress that influences the reproductive hormones' impairment.

### Histological assessment on the TQ protective effect on AA-induced ovarian injury

**Figure 4:**
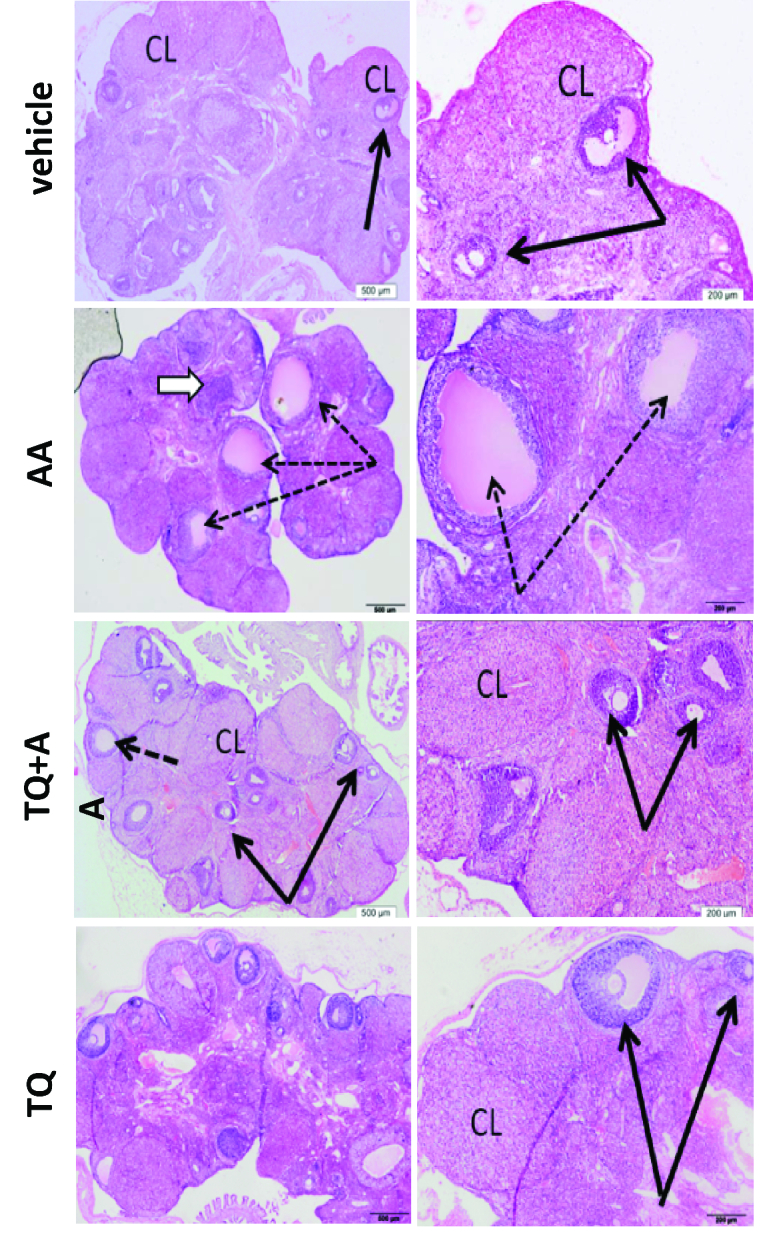
Effect of thymoquinone (TQ) on female rat ovary aberrations induced by acrylamide (AA). The vehicle group showed normal follicles (black arrow) with intact ova and numerous normal corpora lutea (CL). AA group showed cystic changes in developing follicles (dotted arrows). Corpus luteum showed degenerated cells with dark nuclei (CL). AA+TQ group showed preservation of follicular structures (black arrows), residual small cysts are still present (dotted arrow). TQ group showed numerous follicles (arrows) with normal structure, and intact oocytes (black arrows), Corpus luteum (CL) showed normal, more healthy features than vehicle (H&E and E x400).

In the present study, histological examination of ovarian tissue showed the enhancing effect of TQ on AA-intoxicated ovaries tissue. The AA group showed obvious ovary damage including multiple cysts, absence of mature follicles, and disorganized degenerated corpora lutea. TQ+AA group was found to preserve the normal structure of rat ovaries and nearly normal follicles and corpora similar to the vehicle group. The TQ group did not alter the normal structure of the ovary and even looked more organized compared to the vehicle (Figure 4).

#### Quantitative analysis of different ovarian structures in vehicle, TQ, AA, and TQ+AA groups

Table I showed that AA administration result in statistically significant decrease in all types of ovarian follicles and corpora lutea with a significant increase in cystic or atretic changes. On the other hand, administration of TQ to AA-treated group showed significant preservation of ovarian structures quantitative parameters compared to non-treated group. This analysis provided support for the histological observation demonstrated in hematoxylin and eosin-stained histological sections.

### Computational results

**Figure 5:**
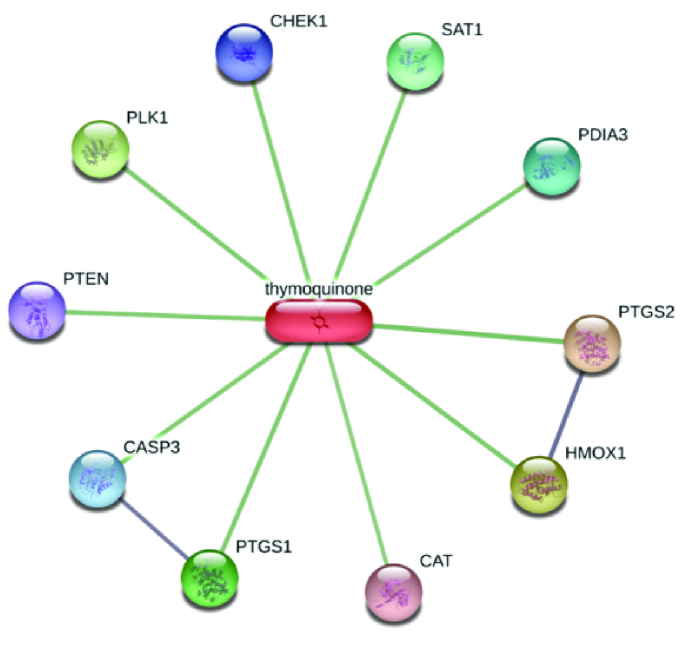
Prediction of the target interacting proteins with TQ. Protein-protein interactions are shown in grey and protein-drug interactions are shown in green.

**Figure 6:**
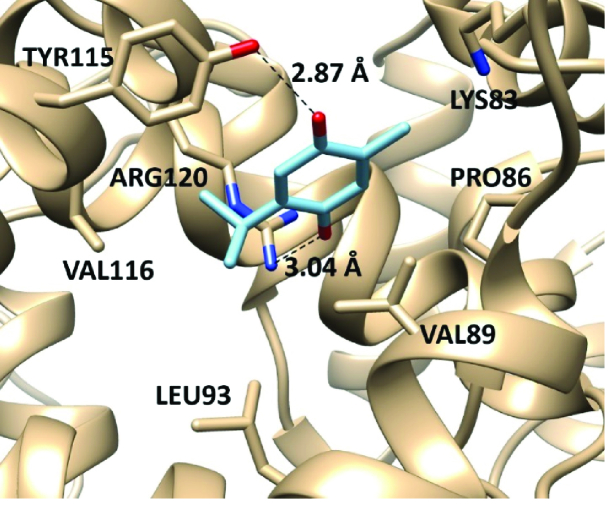
Molecular interactions of TQ with the target protein COX-2. TQ forms 2 hydrogen bonds with the side chain of Y115 and R120. Also, it showed a hydrophobic interaction with the hydrophobic residues in the active site of COX-2.

Predicting the target proteins for TQ was performed, and network analysis was constructed as shown in figure 5. The model comprises 10 nodes and 11 edges, and the scoring data of all the predicted protein interactions with TQ were listed in table II. The prediction output showed that prostaglandin-endoperoxide synthase 2 (prostaglandin G/H synthase and cyclooxygenase) (COX-2) has the highest potentiality to interact with TQ with a scoring function of 0.883. Therefore, a molecular docking approach was employed to assess the affinity of TQ with COX-2. We utilized the molecular docking approach for interpreting the molecular mechanism of the anti-inflammatory activity of TQ as a potentially safe and effective COX-2 inhibitor. TQ exhibited a promising binding affinity with COX-2 with docking energy of binding -6.12 ± 0.48 kcal/mol. TQ exhibited various non-covalent interactions with the active site of COX-2. It makes 2 hydrogen bonds with the side chain of Y115 and R120 and exhibited a hydrophobic interaction with V89, L93, and V116 in the binding pocket of COX-2 (Figure 6).

**Table 1:** Statistical quantitative analysis of folliculogenesis in control vs. different experimental groups


**Parameter**	**Vehicle**	**AA**	**AA+TQ**	**TQ**
**“Primordial follicles"**	21.80 ± 0.9695	5.500 ± 0.5000 ${}^{***}$	17.10 ± 0.8426 ${}^{*}$	18.46 ± 1.628 ${}^{*}$
**“Growing follicles"**	9.700 ± 0.8888	2.100 ± 0.5568 ${}^{***}$	5.500 ± 0.7416 ${}^{**}$	8.460 ± 0.5016 ${}^{NS}$
**“Mature gaffe follicles"**	7.960 ± 0.8376	1.800 ± 0.5831 ${}^{***}$	7.200 ± 0.6633 ${}^{NS}$	6.660 ± 1.023 ${}^{NS}$
**“Corporal luteal"**	8.320 ± 1.021	4.820 ± 0.5122 ${}^{*}$	6.560 ± 0.8298 ${}^{NS}$	8.560 ± 0.8298 ${}^{NS}$
**Cystic or atretic follicles**	0.5000 ± 0.2236	7.200 ± 0.8602 ${}^{***}$	2.700 ± 0.8307 ${}^{NS}$	1.900 ± 0.5099 ${}^{NS}$
Findings are offered as Mean ± SE (n = 4). Significant against. *P < 0.05, **P < 0.01, ***P < 0.001, NS: Not statistically significant, TQ: Thymoquinone, AA: Acrylamide				

**Table 2:** Main predicted target information


**#Node1**	**Node2**	**Node1_string_internal_id**	**Node2_string_internal_id**	**Combined_score**
**Prostaglandin-endoperoxide** **synthase 2**	Thymoquinone	1795390	-100010281	0.883
**Heme oxygenase 1 **	Thymoquinone	1789138	-100010281	0.821
**Polo-like kinase 1 **	Thymoquinone	1791523	-100010281	0.815
**Prostaglandin-endoperoxide** **synthase 1 **	Thymoquinone	1788934	-100010281	0.809
**Caspase 3, apoptosis-related** **cysteine peptidase **	Thymoquinone	1798025	-100010281	0.795
**Protein disulfide isomerase family A **	Thymoquinone	1800749	-100010281	0.795
**Phosphatase and tensin homolog **	Thymoquinone	1782620	-100010281	0.795
**Checkpoint kinase 1 **	Thymoquinone	1787000	-100010281	0.795
**Spermidine/spermine** **N1-acetyltransferase 1 **	Thymoquinone	1801834	-100010281	0.795
**Prostaglandin-endoperoxide** **synthase 2 **	Heme oxygenase 1	1795390	1789138	0.789
**Catalase **	Thymoquinone	1789877	-100010281	0.737
**Caspase 3, apoptosis-related** **cysteine peptidase **	Prostaglandin-endoperoxide synthase 1	1798025	1788934	0.709
The prediction scores of drug-proteins and protein-protein interactions were generated from http://stitch.embl.de/				

## 4. Discussion

This study was conducted to evaluate the potential therapeutic effect of TQ on the reproductive impairment induced by AA in female rats. AA administration caused severe impairment in the reproductive system at the hormones and histological levels. AA significantly reduced estradiol and progesterone levels. Also, it increases the LH levels but no significant changes in FSH levels.

Similar findings have been observed in previous studies (30). These findings could be attributed to the serious reduction in corpus lutea in AA-administrated rats. The current data showed the carcinogenic effect caused by AA administration. AA significantly increases the levels of CA125 and CEA as tumor biomarkers. AA is classified by the International Agency for Research on Cancer as a probable human carcinogen. These carcinogenic effects are due to the ability of AA and its epoxide metabolite glycidamide to form DNA adduct (6). Also, AA reacts with glutathione and disturbs the redox status of cells and gene transcription and hormonal balances (31). In addition, AA administration exhibits a deleterious effect on cells' oxidative stress, leading to reproductive impairment. AA significantly increases TBARS levels and reduces the catalase and TAC contents. Similar findings have been reported previously (32).

Furthermore, histological assessment showed the absence of mature follicles and disorganized degenerated corpora lutea in AA-treated rats. Similar data have been reported previously (33). These results suggest that AA affects corpus luteum formation and follicular development. The histological observation reported in this study was further supported by statistical quantitative analysis of different ovarian elements in both AA and TQ-treated groups compared to control and non-treated rats. In the present study, TQ attenuated AA-induced reproductive impairment and oxidative stress in female rats. The antioxidant efficacy of TQ has been previously reported in some studies (14, 34). The ameliorative effect of TQ in the reproductive system is attributed to its great antioxidant activity that attenuates the redox imbalance in AA-treated rats.

Additionally, inflammation is one of the immune responses during pathogenesis, cancer progression, infection, and injuries. Also, inflammation has a crucial effect on fertility suppression because it can disturb the function of gonadotropin-releasing hormone neurons, which is the primary regulator of fertility in females (35). Cyclooxygenases are the primary enzymes that regulate and modulate the inflammation process. COX-1 and COX-2 are the 2 isoforms of cyclooxygenase. COX-1 maintains various homeostatic functions and is expressed throughout the body, while COX-2 is involved in the generation of prostaglandins that mediate the inflammation process and COX-2 is only expressed during the inflammation (36). COX-1 and COX-2 are the main targets for nonsteroidal anti-inflammatory drugs (NSAIDs) (37). In addition, NSAIDs increase estrogenicity and cause several adverse effects on reproduction (38).

On the other hand, TQ exhibited extraordinary anti-inflammatory and immunomodulatory effects as one of the major bioactive components of *Nigella sativa *seeds extract. Interestingly, the network protein-drug interaction model showed that TQ has the highest affinity to COX-2 and strongly suggested its inhibitory effect on COX-2 activity and its anti-inflammatory role. Based on the constructed protein-drug model, COX-2 was the most interesting target for TQ. The docking energy of binding of TQ with COX-2 (-6.12 ± 0.48 kcal/mol) is relatively close to the value obtained for standard COX-2 inhibitor SC-558 (-6.86 ± 0.33 kcal/mol) (39). This finding emphasizes the importance of studying the protective effect of TQ on the reproductive system against AA-induced toxicity as a safe and efficient alternative for NSAIDs.

TQ showed remarkable anti-inflammatory, antioxidant, and protective effects on ovarian health against ischemia/reperfusion injury (40). Also, TQ ameliorates fertility disorders induced by oxidative stress at the steroidal hormone levels and tissue structure (41). These findings push us to study the protective effect of TQ against AA-induced reproductive toxicity. Our findings showed the protective effect of TQ on ovarian health and oxidative stress levels induced by AA administration. In addition, we utilized different bioinformatics approaches to investigate the potential molecular mechanism beyond the protective effect of TQ through the interaction with its suggested molecular target COX-2 the main modulator of inflammation. However, experimental binding data between TQ and COX-2 is required to verify our molecular docking findings.

## 5. Conclusion

In conclusion, TQ reduced oxidative stress and enhanced reproductive hormone levels. Also, TQ administration preserves the healthy ovary structure. *In silico* analyses are strongly suggested that TQ could be a COX-2 inhibitor, this finding may also be exploited as a safe alternative for NSAIDs. Our data showed that TQ could be used as a safe agent to protect humans and/or animals against the adverse health effects of AA.

##  Conflict of Interest 

The authors declare that there is no conflict of interest.
